# Enhanced GNSS Reliability on High-Dynamic Platforms: A Comparative Study of Multi-Frequency, Multi-Constellation Signals in Jamming Environments

**DOI:** 10.3390/s23239552

**Published:** 2023-12-01

**Authors:** Abdelsatar Elmezayen, Malek Karaim, Haidy Elghamrawy, Aboelmagd Noureldin

**Affiliations:** 1 Electrical and Computer Engineering Department, Royal Military College of Canada, Kingston, ON K7K 7B4, Canada; malek.karaim@queensu.ca (M.K.); haidy.elghamrawy@rmc.ca (H.E.); aboelmagd.noureldin@rmc.ca (A.N.); 2 Public Works Engineering Department, Tanta University, Tanta 31552, Egypt; 3 Electrical and Computer Engineering Department, Queen’s University, Kingston, ON K7L 3N6, Canada

**Keywords:** GNSS, jamming, multi-constellation, multi-frequency, interference, navigation and positioning, autonomous vehicles

## Abstract

The global navigation satellite system (GNSS) signals are vulnerable to disruption sources, such as signal jamming. This, in turn, can cause severe degradation or discontinuities of the GNSS-based position, navigation, and timing services. The availability of multi-frequency signals from multi-constellation GNSS systems, such as Galileo and GLONASS, along with the modernization of GPS with multi-frequency signals, has the potential to increase the immunity of GNSS-based navigation systems to signal jamming. Despite various studies completed on the utilization of multi-frequency and multi-constellation global navigation satellite system (GNSS) signals to resist receiver jamming, there is still an urge to further investigate this concern under different circumstances. This paper presents an experimental evaluation of the advantages of the employment of multi-frequency multi-constellation GNSS signals for better GNSS receivers’ performance during signal jamming situations for high-dynamic platforms such as aircraft/drones. Additionally, the study examines the effects of both simulated and real jamming signals on all possible combinations of the GPS, Galileo, and GLONASS signal frequencies and constellations. Two airplane trajectory routes were built, and their corresponding RF signals were generated using the Spirent and Orolia GNSS signal simulators. The results indicated that the GPS multi-frequency-based solution maintains reliable positioning performance to some extent under low jamming scenarios. However, the combination of GPS, Galileo, and GLONASS signals proved its ability to provide a continuous and accurate positioning solution during both low and high jamming scenarios.

## 1. Introduction

The need for precise and reliable positioning and navigation services is becoming a critical requirement, particularly in applications such as autonomous land and air vehicles [1,2,3,4]. For instance, the safety of passengers, pedestrians, and cargo becomes a paramount concern with the use of autonomous land and air vehicles. This, in turn, requires reliable and continuous positioning and navigation solutions to ensure that these vehicles can accurately perceive their surroundings and make informed decisions to avoid collisions and navigate complex environments efficiently [5,6,7].

In such safety-critical applications, relying solely on single-frequency or single-constellation Global Navigation Satellite System (GNSS) signals to provide positioning solutions can be risky. This is because the GNSS signals are transmitted to users’ receivers with very low signal power, making them susceptible to both unintentional and malicious interference, commonly known as jamming [8,9,10]. The jamming term refers to the deliberate transmission of radio frequency signals on the same frequency as the GNSS signals, overpowering or disrupting the reception of these signals by GNSS receivers [11]. The consequences of jamming in safety-critical applications can be severe, leading to deteriorating positioning accuracy or even complete loss of position information for a large area around the jammer, depending on the inserted jamming signal power [12]. The use of multi-frequency and multi-constellation GNSS signals has the potential to mitigate the effects of jamming and enhance the reliability of the positioning solution. By employing multiple frequencies and signals from different constellations (such as GPS, GLONASS, Galileo, BeiDou, etc.), the receiver gains several advantages. First, this will add high signal redundancy, ensuring that if one signal is jammed or made unavailable, the receiver can still rely on other signals to maintain accurate and continuous positioning. Second, access to multi-frequency signals from different constellations grants the receiver a higher chance of receiving more useful signals; thus, enhancing the received signal strength and hence improving the positioning accuracy and availability. Third, a receiver capable of tracking several signals from various frequencies and constellations can effectively identify anomalies in signal behavior, enabling the detection and mitigation of jamming signals.

The objective of this paper is to extensively explore the advantages of using multi-frequency multi-constellation GNSS over GPS-only systems regarding signal jamming resistance and positioning solution availability. To achieve this goal, two trajectories are simulated using the Orolia GNSS simulation engine. The simulated data are then either altered by an internally simulated interference signal or hit by a real jamming signal generated by the NavWar Electronic Attack Trainer (NEAT) jammer. These jamming tests took place within the controlled environment of an anechoic chamber room at the Royal Military College of Canada (RMCC). The output trajectory data are then collected using the NovAtel PwrPak7 GNSS receiver. The rest of the paper is organized as follows: Section 2 summarizes relevant research and findings. Detailed methodologies used in this research work are depicted in Section 3. Section 4 describes the experiment setup components, trajectory data logging, and applied jamming scenarios. Results discussions are split over Section 5 and Section 6, where the former is dedicated to the simulated jamming results and the latter to the actual jamming results analysis. Further discussion of all test results is given in Section 7. Section 8 wraps up the paper with conclusions and remarks about the research findings.

## 2. Literature Review

Extensive research in the literature has thoroughly investigated the advantages of utilizing multi-frequency and multi-constellation signals [13,14,15,16]. In [17], the authors carried out several experiments to evaluate the performance of a high-end commercial receiver under diverse jamming conditions, including varying the jamming signal strength and type. They replicated the same trajectories twice: Once with only the GPS signal enabled and once with both GPS and GLONASS constellations enabled. Furthermore, the paper investigated the effects of jamming on both the GPS and GLONASS bands. The findings suggest that employing multi-constellation systems offers a promising solution to mitigate jamming issues, particularly in terms of solution availability and reliability. In [18], additional GNSS constellations and frequencies beyond GPS L1 are usable for automotive applications by including them in an integrated GNSS/INS localization algorithm. The results showed that the main advantages are increased satellite availability, the elimination of ionospheric error, and reduced vulnerability to high-power interference signals. The authors in [19] explored the benefits of the use of multi-constellation on high-dynamic platforms experiencing jamming. Several evaluation metrics were used to evaluate the performance, including satellite availability, carrier-to-noise ratio (C/N0), dilution of precision (DOP), the percentage of solution availability, the minimum number of available satellites, and positioning accuracy. The comparison between using GPS-only and combined GPS/GLONASS revealed that the latter yielded superior results across all evaluation metrics used. In [20], the utilization of multi-constellation receivers in combination with interference mitigation techniques has demonstrated enhancements in both position availability and accuracy. The authors in [21] examined the susceptibility of existing maritime receivers to jamming and explored whether enhancing system resilience could be achieved by upgrading to a multi-constellation or multi-frequency receiver. The results showed that single-frequency multi-constellation receivers offered better jamming robustness when compared with multi-frequency (L1 + L2) GPS receivers. Another important finding is that the GLONASS constellation showed better robustness against jamming as compared to the GPS constellation. The results also suggest dependence on GPS L1 signals in the acquisition of GPS L2 signals. With these findings, the authors suggested the use of multi-constellation GPS/GLONASS receivers as the most economical solution for enhancing jamming robustness. The authors in [22] examined the effectiveness of a software-defined GNSS receiver that operates on multi-frequency, multi-constellation signals. They conducted this analysis both in the presence and absence of a commercial L1 jamming signal. They studied the potential advantages associated with utilizing multi-constellation and multi-frequency approaches to enhance their capabilities for mitigating jamming effects. The results showed that the presence of multiple signals and constellations would result in enhanced positioning availability and reliability; however, it would have a minimal effect on positioning accuracy except in challenging signal conditions, such as the presence of a jamming signal. Based on these findings, the authors suggested assessing the satellite signal quality from various GNSS constellations before incorporating them into a combined navigation solution.

## 3. Methodology

In this study, the positioning performance of the GNSS receiver was evaluated in both simulated and real signal-jamming environments. A continuous wave (CW) jamming signal was applied on the L1 frequency (1575.42 MHz) at low- and high-power levels for both jamming conditions. For the simulated jamming, the jamming signal power level was 46 dB compared to the reference signal power (−130 dBm) for the low-power level. On the other hand, the power level of the jamming signal was set to 60 dB for the high-power level. To exert the low and high-power jamming levels for the actual jamming condition, a reference signal power of −130 dBm was used. After that, signal attenuations of 36 dB and 20 dB were applied to provide the low and high-power jamming levels, respectively. The jamming signal lasted for two minutes, and it was applied during a portion of the trajectory where the platform was experiencing low dynamics to analyze the effect of the jamming only. The GNSS receiver positioning performance was assessed in three consecutive steps. First, the contribution of using GPS multi-frequency measurements to the resulting positioning solution under both low and high jamming was evaluated. The benefit of GPS multi-frequency measurements was explored by enabling a dual-frequency (DF) signal at a time and then enabling GPS triple-frequency (TF) signals. Second, the benefit of multi-constellation to the GNSS receiver performance is assessed by enabling GPS TF signals and Galileo single-frequency (SF) signals at a time, then enabling GPS TF signals and Galileo TF signals all at once. Lastly, the GNSS receiver was assessed by enabling GPS TF, Galileo TF, and GLONASS SF signals at a time and then enabling GPS TF signals, Galileo TF signals, and GLONASS DF signals all at once. This, in turn, has the capability of providing improved performance due to the high number of visible Galileo and GLONASS satellites when there is no availability of GPS satellites. The used GNSS signal combinations are presented in detail in Table 1.

## 4. Experiment Setup and Data Collection

**Figure 1:**
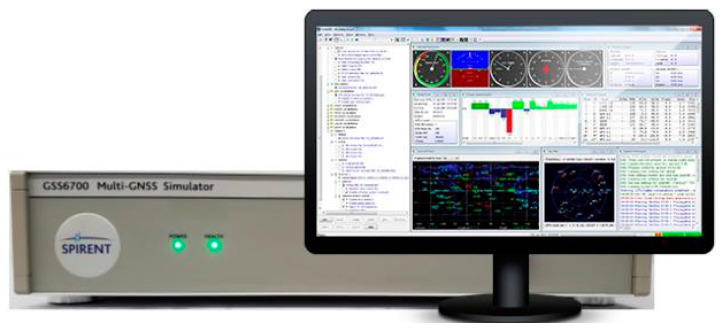
Spirent GSS6700 GNSS simulator.

In this study, two simulated aircraft trajectories were generated. The trajectories present a high range of dynamics, including but not limited to accelerating to a velocity greater than 200 m/s, deaccelerating, sharp turns, a 300 m to 600 m height change, and a 90° heading change. The trajectories were simulated to start at latitude 45°54.744′, longitude −77°17.364′, and altitude 100 m on 30 August 2022, midnight. Firstly, the Spirent GSS6700 GNSS simulator, as indicated in Figure 1, was used to simulate the aircraft trajectories due to its ability to simulate high-dynamic vehicle trajectories.

**Figure 2:**
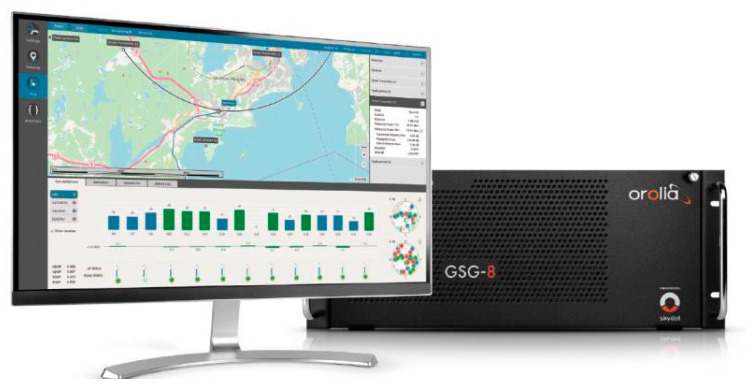
Orolia GSG-8 GNSS simulator.

**Figure 3:**
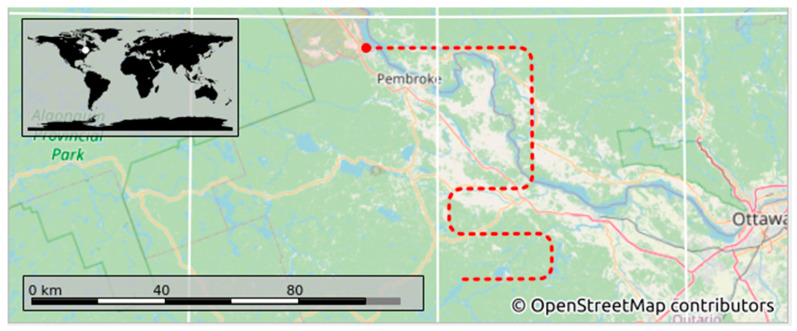
Imported trajectory to Orolia.

The simulated trajectories were then imported to the Orolia GSG-8 GNSS simulator, as shown in Figure 2, due to its ability to simulate multi-frequency, multi-constellation GNSS signals. An overview of the imported trajectory to the Orolia simulator is presented as indicated in Figure 3, for further check before starting the simulation experiments.

**Figure 4:**
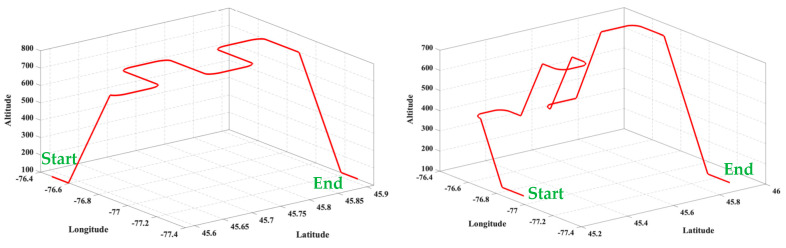
Simulated trajectories: (**left**) first trajectory, (**right**) second trajectory.

The 3D views of the simulated aircraft trajectories are presented in Figure 4. The first trajectory extended for approximately 27 min, with data logging commencing after 2 min from the start, resulting in 25 min of logged data. The second trajectory extended for approximately 24 min, with data logging commencing after 2 min from the start, resulting in 22 min of logged data. The Orolia GNSS simulator’s output was then used for simulated and actual jamming experiments, as described in the following sections.

### 4.1. Simulated Jamming Experiment

**Figure 5:**
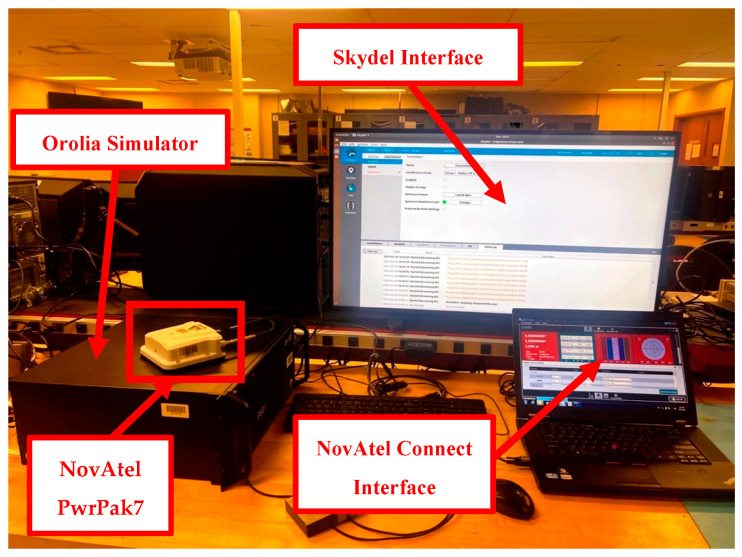
Orolia experimental setup.

The main component of the experiment is the Orolia GSG-8 GNSS signal simulator, driven by the Skydel software version 23.8. Figure 5 shows a general view of the simulation system. The GNSS experiment configuration consists of the Orolia GSG-8 GNSS signal simulator connected to a desktop computer hosting the simulation software (Skydel). The Orolia GSG-8 simulator generates RF signals for multi-frequency, multi-constellation scenarios that are created through Skydel software. The Skydel simulation engine is designed to reproduce a range of satellite constellations, realistic conditions, and even interference attacks [23]. Subsequently, as depicted in Figure 5, the RF output from the simulator is linked to the NovAtel PwrPak7 receiver, and data are captured using the NovAtel connect, which is then processed and analyzed. The NovAtel PwrPak7 is used due to its capability of tracking all GNSS constellations and signals. It has an interference toolkit that enhances its capability for interference detection and mitigation [24]. This capability is deactivated for the whole study to assess the effect of using multi-frequency, multi-constellation GNSS signals for better performance under interference conditions. For simulated jamming, the Skydel is used to control the insertion of the signal along with the jamming signal power. It is worth mentioning that the jamming signal was applied only on L1 frequency due to the limitations of the Orolia simulator output radios used to transmit the output RF signals. In the Orolia GSG-8, there are four radios that can be used to transmit GPS TF signals, Galileo TF signals, and GLONASS DF signals, in addition to only one jamming signal.

### 4.2. Actual Jamming Experiment

**Figure 6:**
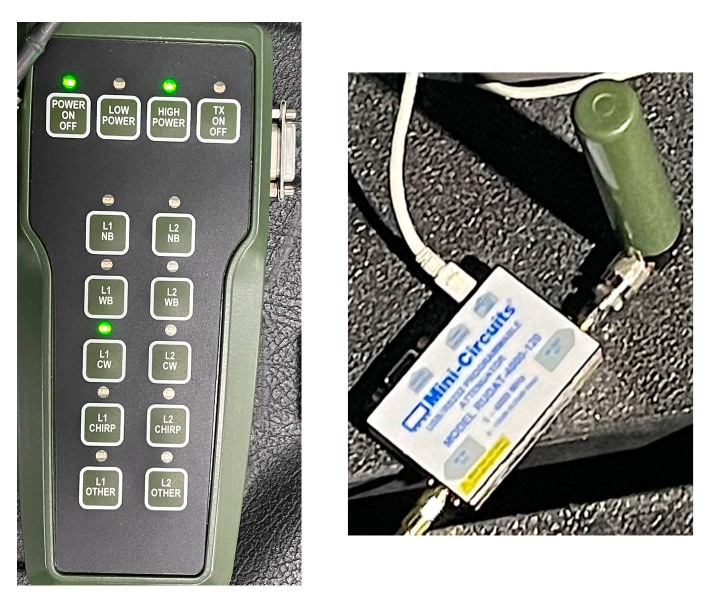
NEAT (**left**), and mini-circuits attenuator (**right**).

**Figure 7:**
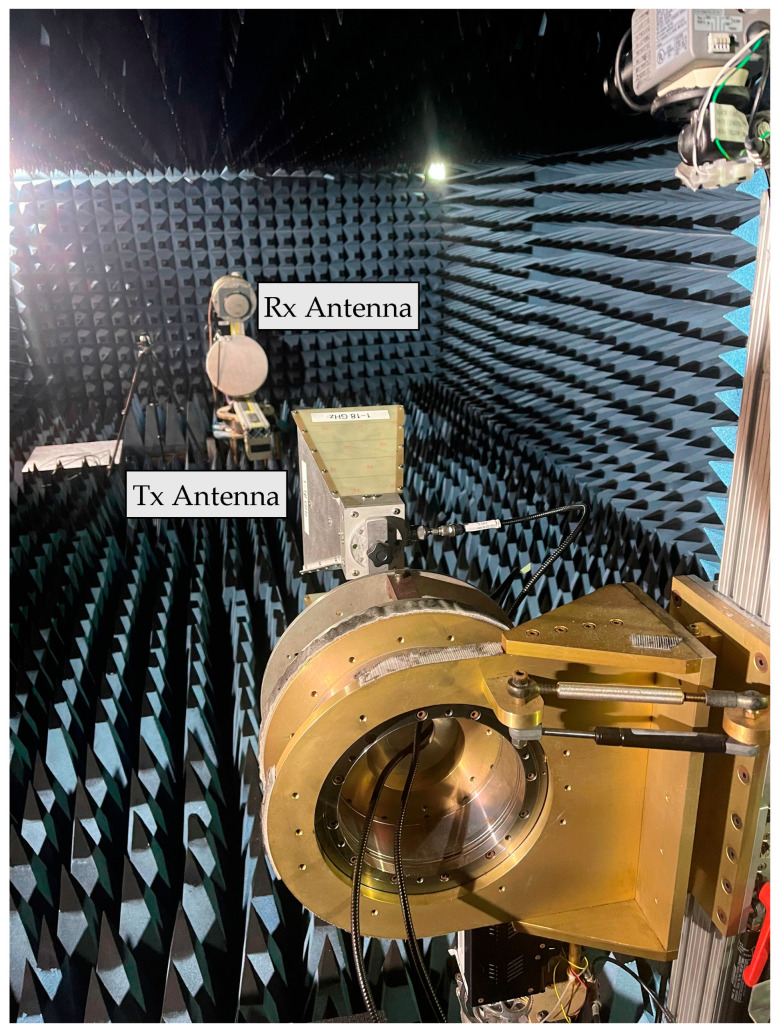
Anechoic chamber including both Tx and Rx antennas.

To apply jamming in this experiment, a NavWar Electronic Attack Trainer (NEAT) jammer was used to generate a real jamming signal inside an anechoic chamber. The NEAT has the capability to generate signal jamming on L1 and L2 frequencies with two levels of jamming power, namely low and high levels. Additionally, it can generate different jamming signals, such as continuous wave, chirp, wide-band, and narrow-band. For the sake of consistency, the jamming signal was applied only for L1 frequency, as in the simulated jamming case. Additionally, a programmable attenuator by Mini-Circuits was connected to the NEAT to control the jamming signal power level inserted inside the anechoic chamber. The NEAT, the mini-circuits attenuator connected to the NEAT antenna, and the mini-circuits attenuator interface are shown in Figure 6. The Orolia GSG-8 GNSS signal generator is still the main component of this experiment. However, the RF signal generated from the Orolia is transmitted to an actual GNSS antenna (Rx antenna) using a radio transmitter (Tx antenna) inside the anechoic chamber, as depicted in Figure 7. The antenna is then linked to the NovAtel PwrPak7 receiver, and data are captured using the NovAtel connect, which is the same as in the simulated experiment.

## 5. Simulated Jamming Results Analysis

The performance of the GNSS receiver is assessed based on its ability to provide a reliable positioning solution and track GNSS satellites under both low and high jamming conditions. The reference solution was logged using the Orolia GSG-8 GNSS simulator. The jamming signal was inserted from minute 14 to minute 16 on the logged data for both the low and high jamming conditions. The timing interval of the jamming signal insertion was chosen to be away from high dynamics up to a certain extent to make sure that the effect on the receiver performance mainly results from the impact of the jamming signal.

### 5.1. Low Jamming Scenario

Firstly, the performance of the GNSS receiver was assessed under low jamming conditions for both the first and second trajectory. It is worth mentioning that the positioning solution is extracted directly from the NovAtel PwrPak7 GNSS receiver without any further processing.

#### 5.1.1. First Trajectory

**Figure 8:**
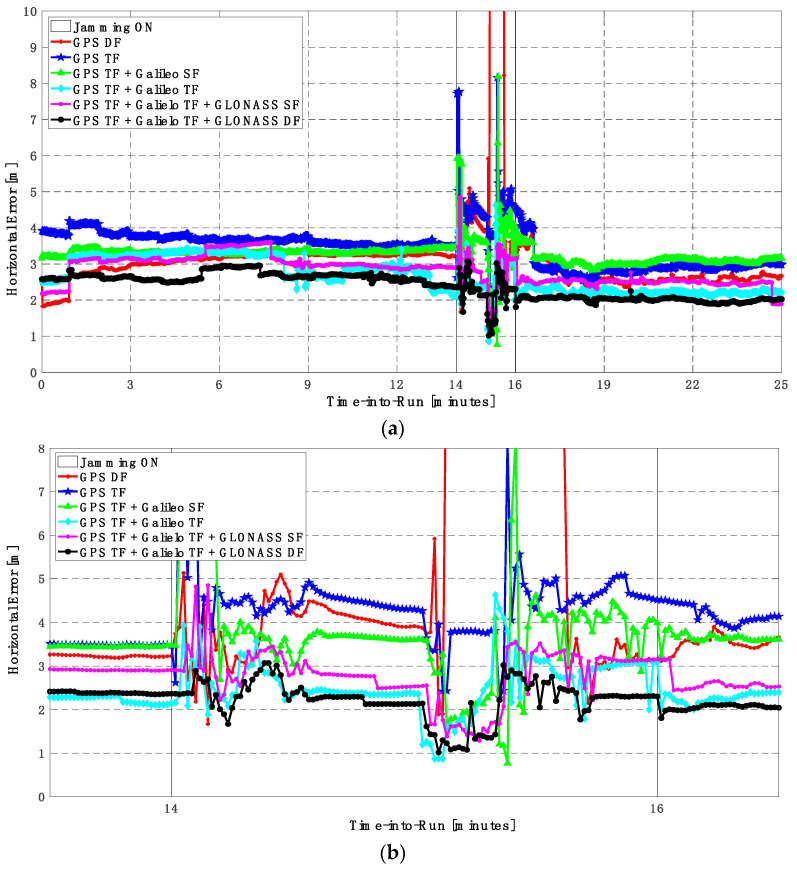
(**a**) Horizontal positioning error for different GNSS signal combinations [first trajectory]. (**b**) Zoomed view of horizontal positioning error for different GNSS signal combinations [first trajectory].

**Figure 9:**
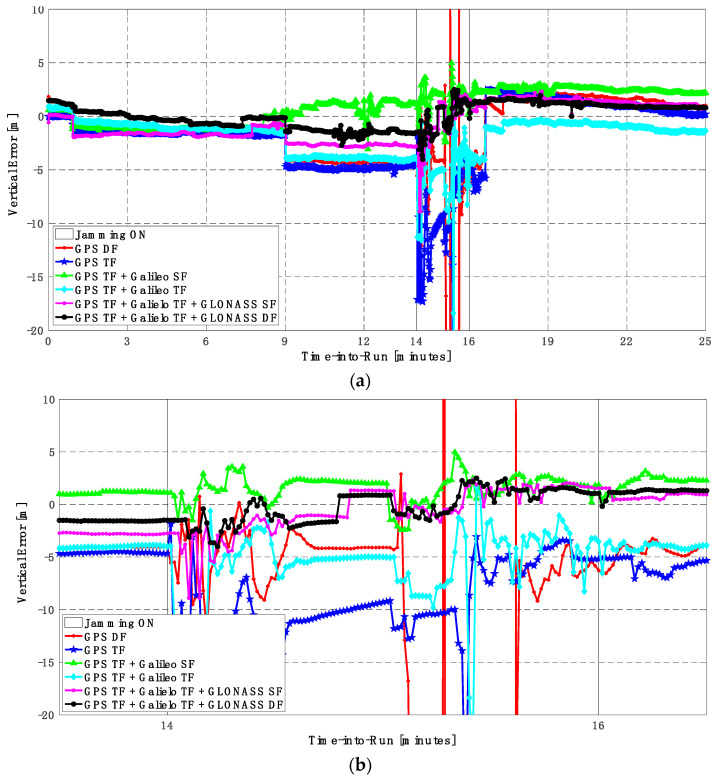
(**a**) Vertical positioning error for different GNSS signal combinations [first trajectory]. (**b**) Zoomed view of vertical positioning error for different GNSS signal combinations [first trajectory].

It can be seen from Figure 8 and Figure 9 that using only GPS signals, either dual-frequency (DF) or triple-frequency (TF) signals, cannot maintain a reliable poisoning solution during the jamming interval that is indicated as a grey-hatched region. The horizontal positioning error exceeds 10 m in the case of using GPS DF signals and reaches a maximum of 8 m during the jamming interval using the GPS TF signals, as shown in Figure 8a,b. The same behavior is depicted for the vertical positioning error, showing errors exceeding 20 m and 15 m for the GPS DF and GPS TF-based solutions, respectively, as in Figure 9a,b. By adding the Galileo SF signal only to GPS TF signals, the performance is similar to the GPS TF case, as shown in Figure 8 and Figure 9. This is because the jamming is attacking the GPS L1 frequency, which is the same as the Galileo E1 frequency, and adding just E1 signals cannot play a major role in improving the performance during jamming. After adding the Galileo TF signals to the GPS TF signals, the positioning solution is significantly improved. Additionally, positioning errors are less than 4 m for both horizontal and vertical components. Moreover, adding GLONASS SF and DF signals to GPS TF and Galileo TF signals improved the performance of the positioning solution. This led to a smoothed positioning solution, especially during jamming intervals, for the GPS TF + Galileo TF + GLONASS DF solution, with a maximum error of about 3 m and 4 m in the horizontal and vertical directions, respectively.

**Figure 10:**
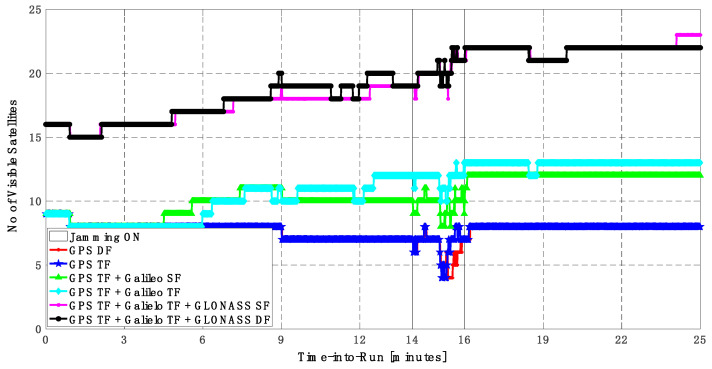
Number of visible satellites for different GNSS signal combinations [first trajectory].

As shown in Figure 10, the number of visible satellites during the jamming period increased from 5 satellites using GPS TF signals to 10 satellites by adding Galileo signals and 20 satellites by adding GLONASS signals, which is reflected in the positioning solution performance as previously indicated.

Table 2 shows the statistical analysis of the first trajectory under low jamming conditions. By using the GPS TF signals, the accuracy of the positioning solution was improved compared to the GPS DF-based solution. The root mean square error (RMSE) is improved by about 23% in the horizontal position component and 55% in the vertical position component, respectively, using GPS TF + Galileo TF signals, compared to using only GPS TF signals. Additionally, using GPS TF + Galileo TF + GLONASS SF signals provides similar positioning accuracy compared to the GPS TF + Galileo TF-based solution. However, by using the GLONASS TF signals instead of GLOANSS SF signals, the RMSE improved by about 11% and 37% in the horizontal and vertical directions, respectively, compared to the GPS TF + Galileo TF-based solution. One more advantage of the inclusion of multi-frequency multi-constellation GNSS signals in the positioning solution estimation is the bounding of the maximum (Max) positioning errors. As shown in Table 2, the horizontal Max error decreased from 1141.527 m using the GPS DF signals to 3.074 m using the GPS TF + Galileo TF + GLONASS DF signals, and the vertical Max error decreased from 3283.053 m using the GPS DF signals to 4.024 m using the GPS TF + Galileo TF + GLONASS DF signals.

#### 5.1.2. Second Trajectory

**Figure 11:**
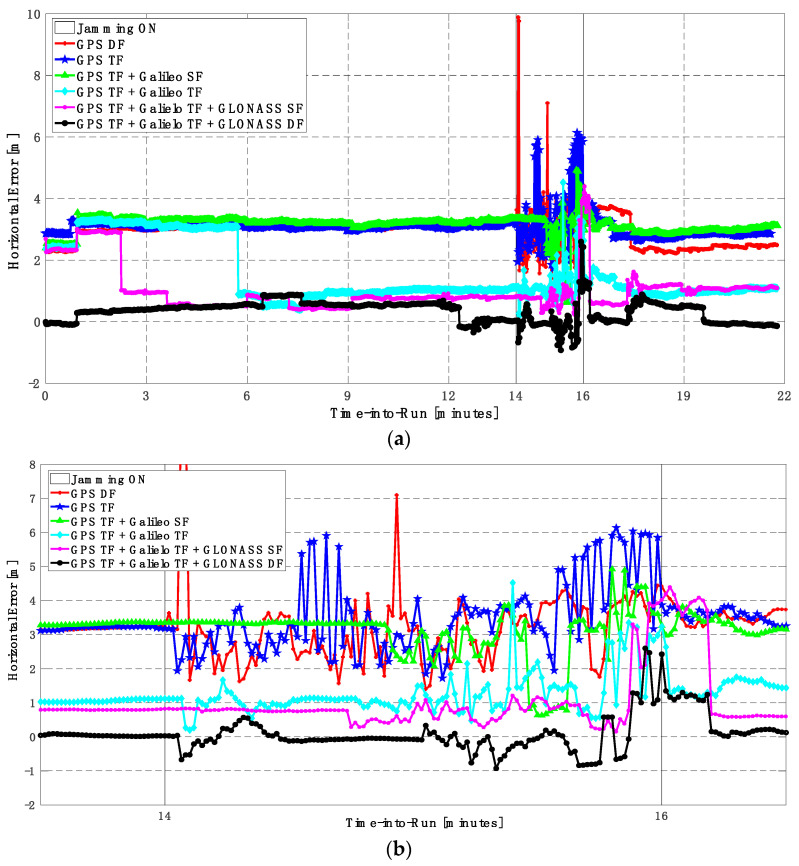
(**a**) Horizontal positioning error for different GNSS signal combinations [second trajectory]. (**b**) Zoomed view of horizontal positioning error for different GNSS signal combinations [second trajectory].

**Figure 12:**
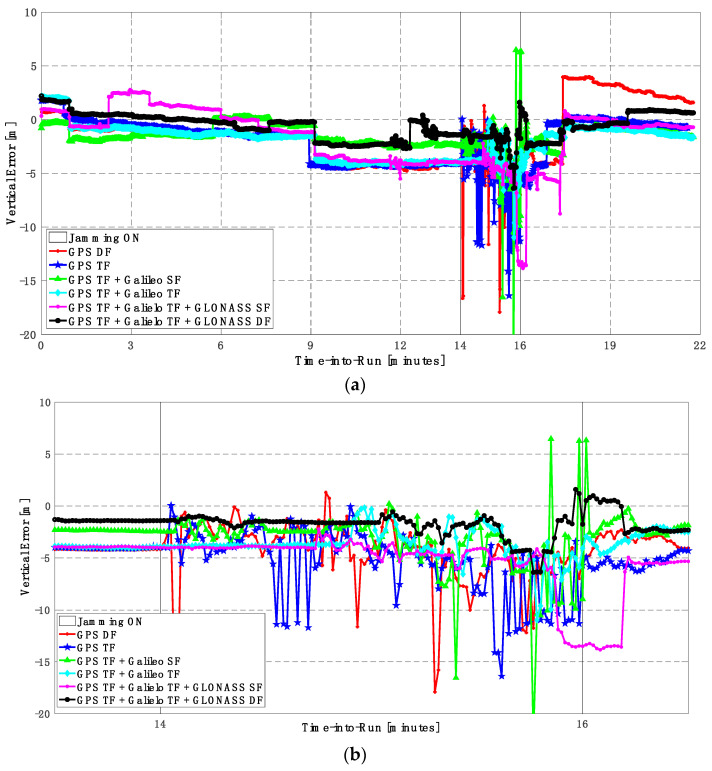
(**a**) Vertical positioning error for different GNSS signal combinations [second trajectory]. (**b**) Zoomed view of vertical positioning error for different GNSS signal combinations [second trajectory].

In this trajectory, the jamming signal was inserted between minutes 14 and 16, as in the first trajectory. Like the first trajectory, by using the GPS DF signals, or the GPS TF signals, the horizontal positioning errors exceed 8 m and 6 m, respectively, as shown in Figure 11a,b. The vertical positioning errors exceed 15 m for both GPS DF and GPS TF-based solutions, as shown in Figure 12a,b. When the Galileo SF signals are added to the GPS TF signals, the horizontal positioning errors are reduced to less than 5 m, but the vertical positioning error still has large spikes during the jamming interval. This is an indicator that adding SF signals from Galileo cannot improve the positioning solution, especially under jamming. Using the Galileo TF along with the GPS TF improves the positioning solution performance under jamming for both the horizontal and vertical positioning components. Additionally, adding the GLONASS SF signals to both the GPS TF and the Galileo TF signals improves the performance of the positioning solution. This leads to a better positioning solution, especially during jamming intervals, for the GPS TF + Galileo TF + GLONASS SF solution, with a maximum error of about 3 m and 10 m in the horizontal and vertical directions, respectively. Moreover, the GPS TF + Galileo TF + GLONASS DF-based solution shows the best performance with horizontal and vertical errors of less than 2 m and 5 m, respectively, during the jamming interval.

**Figure 13:**
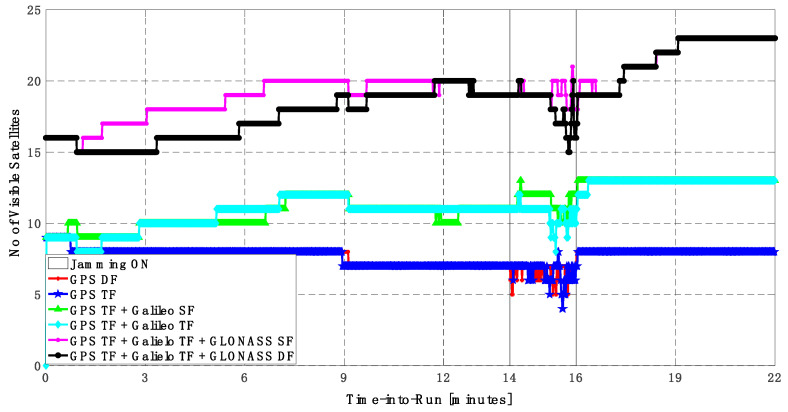
Number of visible satellites for different GNSS signal combinations [second trajectory].

As shown in Figure 13, the minimum number of visible satellites increased from 5 satellites using GPS TF signals to 10 satellites by adding Galileo signals and 15 satellites when adding GLONASS signals. As shown in Table 3, the performance of the GPS TF-based solution is much better than the counterpart solution from the first trajectory. However, the Max positioning errors are about 6 m and 16 m for the horizontal and vertical components of the positioning solution, respectively. Adding the Galileo SF signals does not improve the positioning solution compared to the GPS TF-based solution, as indicated in Figure 11 and Figure 12. However, the root mean square error (RMSE) is improved by about 41% and 30% in the horizontal and vertical directions by using the GPS TF + Galileo TF signals, compared to using only the GPS TF signals. Additionally, the RMSE is improved by about 31% in the horizontal direction by using the GPS TF + Galileo TF + GLONASS SF signals, compared to using only the GPS TF + Galileo TF signals. However, there is unexpected positioning performance of the GPS TF + Galileo TF + GLONASS SF-based solution in the vertical direction, as shown in Figure 12a,b, leading to larger RMSE and Max errors compared to the GPS TF + Galileo TF counterpart. By using the GLONASS TF signals instead of the GLOANSS SF signals, the RMSE is improved by about 73% and 35% in the horizontal and vertical directions, respectively, compared to the GPS TF + Galileo TF-based solution. The maximum horizontal positioning error dropped from 9.882 m by using the GPS DF signals to 2.596 m by using the GPS TF + Galileo TF + GLONASS DF signals, whereas the maximum vertical positioning error dropped from 17.911 m by using the GPS TF signals to 6.393 m by using the GPS TF + Galileo TF + GLONASS DF signals. This is an indicator that using multi-frequency, multi-constellation GNSS signals significantly improves positioning performance in terms of accuracy and reliability.

### 5.2. High Jamming Scenario

The performance of the GNSS receiver was further assessed under high jamming conditions to assess the contribution of using multi-frequency, multi-constellation GNSS signals to improve positioning performance during jamming.

#### 5.2.1. First Trajectory

**Figure 14:**
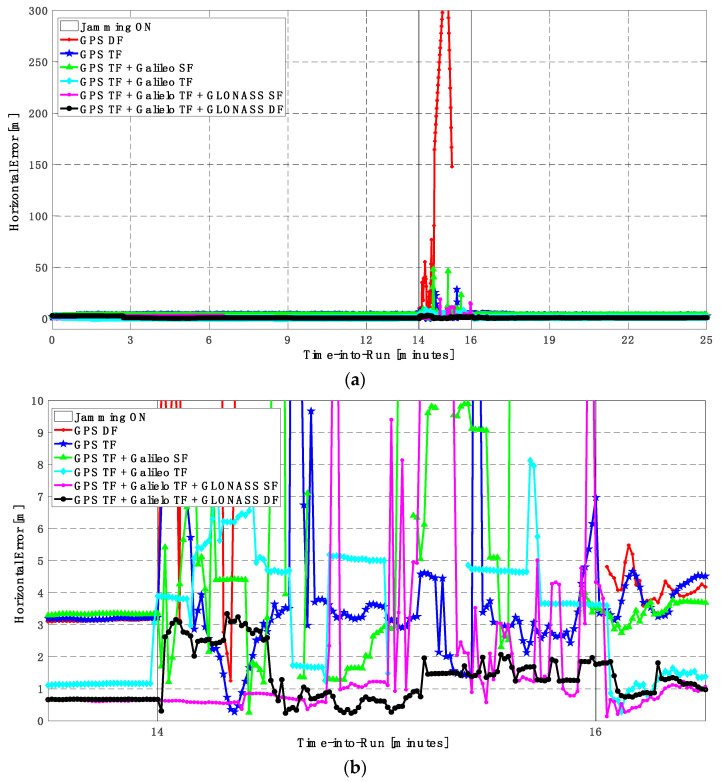
(**a**) Horizontal positioning error for different GNSS signal combinations [first trajectory]. (**b**) Zoomed view of horizontal positioning error for different GNSS signal combinations [first trajectory].

**Figure 15:**
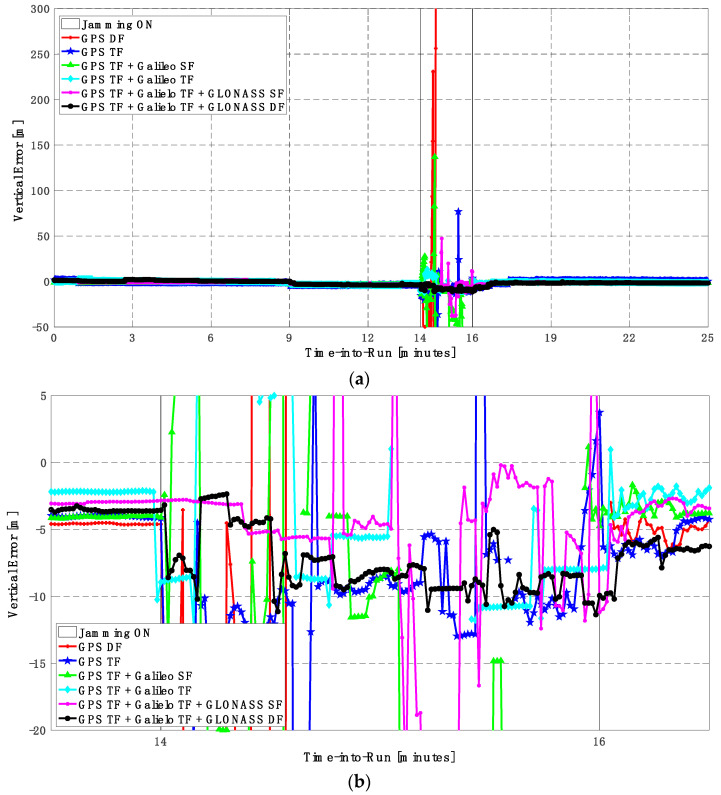
(**a**) Vertical positioning error for different GNSS signal combinations [first trajectory]. (**b**) Zoomed view of vertical positioning error for different GNSS signal combinations [first trajectory].

**Figure 16:**
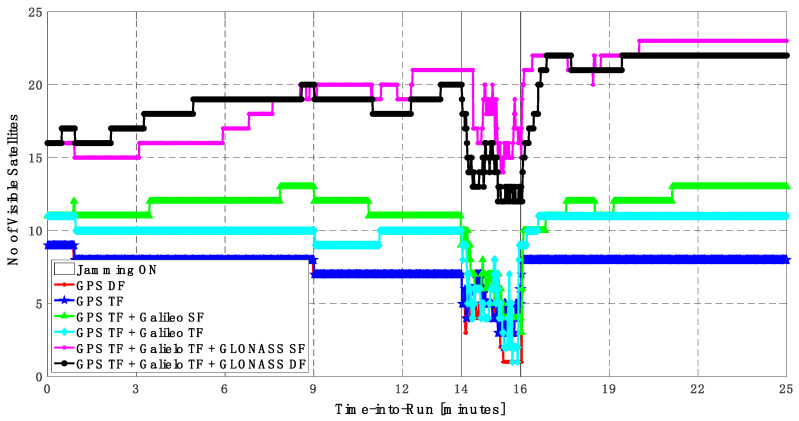
Number of visible satellites for different GNSS signal combinations [first trajectory].

As in the low jamming scenario, the jamming signal was inserted between minutes 14 and 16 of the logged data. With the high jamming, the GPS DF-based positioning solution fails to provide a reliable continuous positioning solution, as indicated in Figure 14 and Figure 15. Additionally, by using the GPS TF signals, the resultant solution has continuous behavior but with large horizontal and vertical positioning errors that exceed 10 m and 20 m, respectively, as shown in Figure 14b and Figure 15b. By using the GPS TF + Galileo SF signals, the positioning solution still suffers from discontinuity during jamming intervals. Additionally, by using the GPS TF + Galileo TF, or the GPS TF + Galileo TF + GLONASS SF, the positioning solution shows continuous behavior. However, it still suffers from large spikes that exceed the 10 m level for both the horizontal and vertical components, respectively, as shown in Figure 14b and Figure 15b. By using all available GNSS signals, namely the GPS TF + Galileo TF + GLONASS DF signals, the positioning solution maintains continuous and reliable positioning performance. As shown in Figure 16, adding the GLONASS signals is the only case that offers a sufficient number of visible GNSS satellites to provide a reliable and continuous positioning solution.

From Table 4, there are three main findings. The former is that adding only the Galileo SF signals to the GPS TF signals does not improve the positioning accuracy due to the high jamming level on the L1 frequency. Second, adding the GLONASS SF to both the GPS TF signals and the Galileo TF does not provide better positioning accuracy compared to using the GPS TF + Galileo TF signals. The latter, using the GPS TF + Galileo TF + GLONASS DF combination, is the only way to have a reliable, accurate, and continuous positioning solution under high jamming. Additionally, the root mean square error (RMSE) is improved by about 52% and 50% in the horizontal and vertical positional directions, respectively, by using the GPS TF + Galileo TF signals, compared to using only the GPS TF signals. Moreover, the RMSE is improved by about 22% in the horizontal positional direction by using the GPS TF + Galileo TF + GLONASS DF signals, compared to using the GPS TF + Galileo TF signals. The maximum horizontal and vertical positioning errors dramatically decreased from 28.611 m and 94.982 m in the case of using the GPS TF signals to 3.347 m and 11.363 m in the case of using the GPS TF + Galileo TF + GLONSS DF signals.

#### 5.2.2. Second Trajectory

**Figure 17:**
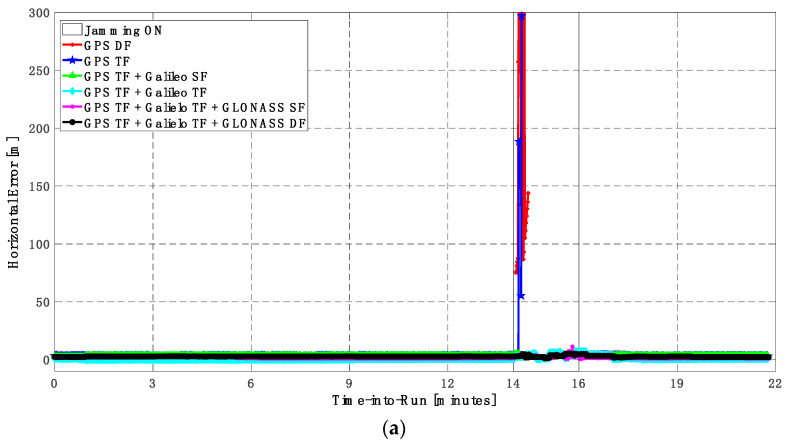

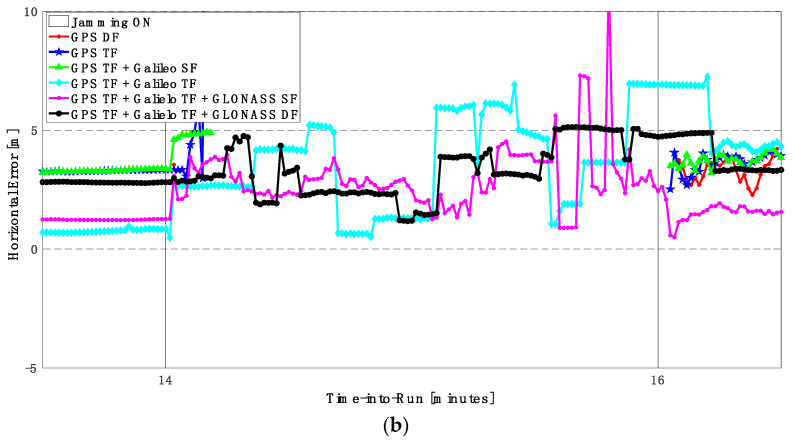
(**a**) Horizontal positioning error for different GNSS signal combinations [second trajectory]. (**b**) Zoomed view of horizontal positioning error for different GNSS signal combinations [second trajectory].

**Figure 18:**
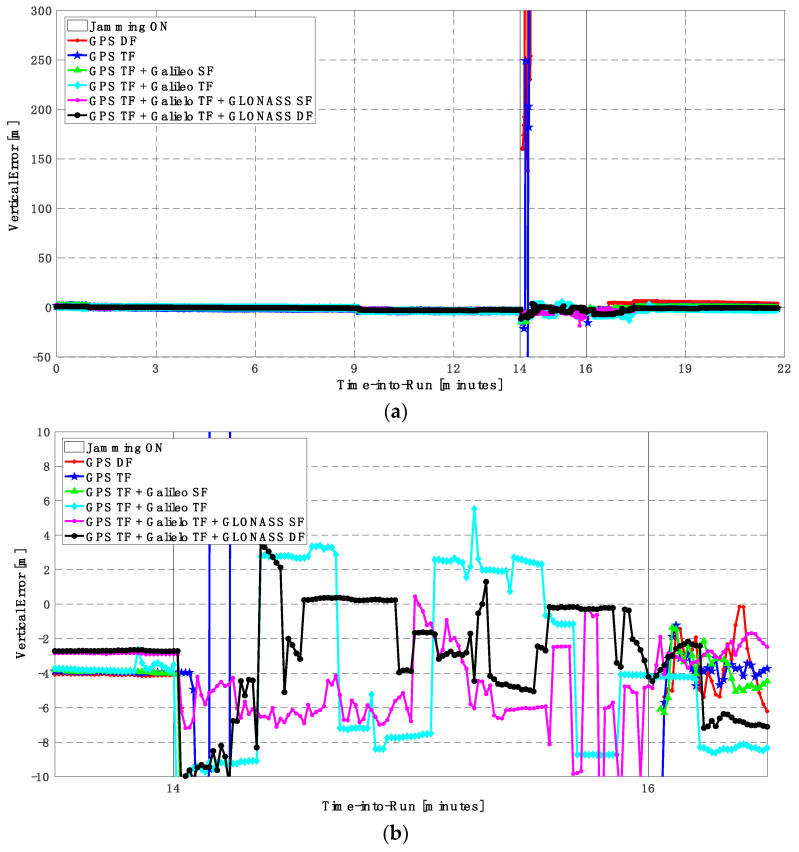
(**a**) Vertical positioning error for different GNSS signal combinations [second trajectory]. (**b**) Zoomed view of vertical positioning error for different GNSS signal combinations [second trajectory].

**Figure 19:**
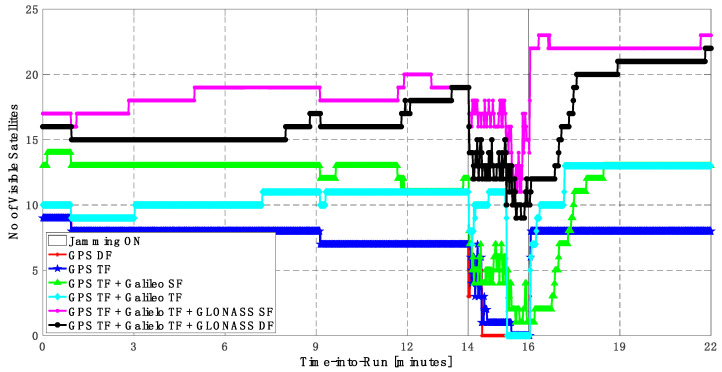
Number of visible satellites for different GNSS signal combinations [second trajectory].

The GPS DF, GPS TF, and GPS TF + Galileo SF-based positioning solutions fail to provide a reliable continuous solution, as indicated in Figure 17 and Figure 18. Additionally, by using the GPS TF + Galileo TF, or the GPS TF + Galileo TF + GLONASS SF, the positioning solutions show continuous behavior. However, there are large spikes for both the horizontal and vertical positioning components, respectively. By using the GPS TF + Galileo TF + GLONASS DF signals, the positioning solution maintains continuous and reliable positioning performance, as shown in Figure 17b and Figure 18b. This is because of the availability of a minimum of 10 satellites during jamming, as shown in Figure 19.

It can be seen from Table 5 that the GPS TF + Galileo SF-based solution provides better accuracy than the GPS TF + Galileo TF counterpart for the vertical component of the positioning solution. However, it is worth mentioning that the GPS TF + Galileo TF solution is available for the whole jamming interval, and the GPS TF + Galileo SF solution is not available for about 90% of the jamming interval. Additionally, it seems that the RMSE of the GPS TF + Galileo TF-based solution is better than the GPS + Galileo TF + GLONASS DF-based solution. However, the latter combination provides a smoothed solution without large spikes, as the maximum horizontal and vertical positioning errors are 5.137 m and 11.658 m compared to 7.245 m and 13.756 m for the GPS TF + Galileo TF solution.

## 6. Real Jamming Results Analysis

In this section, the performance of the GNSS receiver is assessed based on its ability to provide a reliable positioning solution and track GNSS satellites under real jamming conditions. The reference solution was logged using the Orolia GSG-8 GNSS simulator. The jamming signal was inserted from minute 14 to minute 16 on the logged data for both the low and high jamming conditions.

### 6.1. Low Jamming Scenario

Firstly, the performance of the GNSS receiver was assessed under low actual jamming conditions for both the first and second trajectory.

#### 6.1.1. First Trajectory

**Figure 20:**
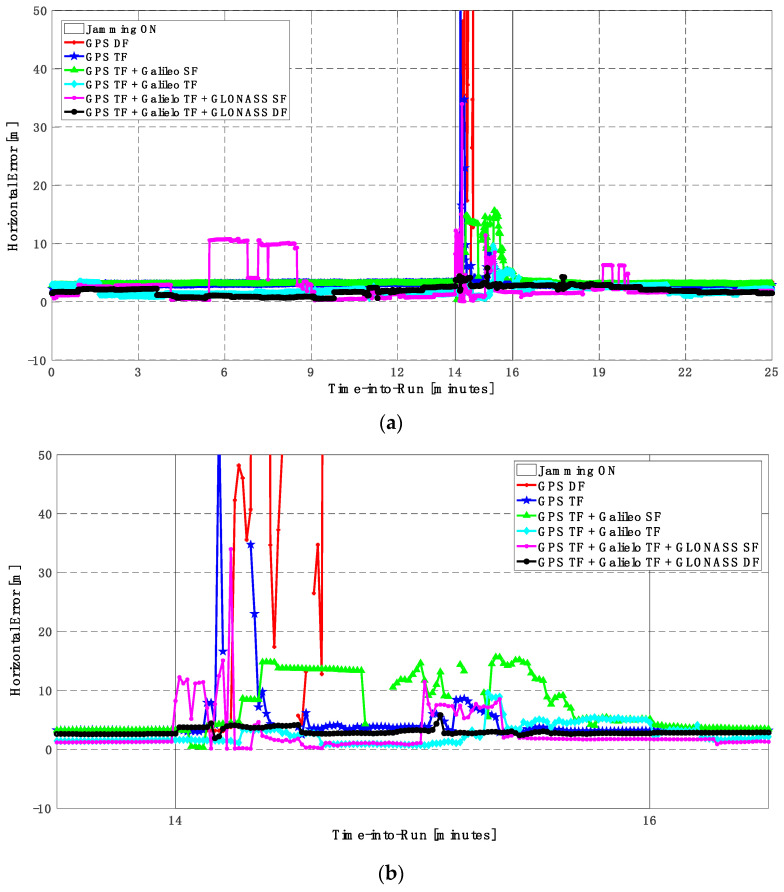
(**a**) Horizontal positioning error for different GNSS signal combinations [first trajectory]. (**b**) Zoomed view of horizontal positioning error for different GNSS signal combinations [first trajectory].

**Figure 21:**
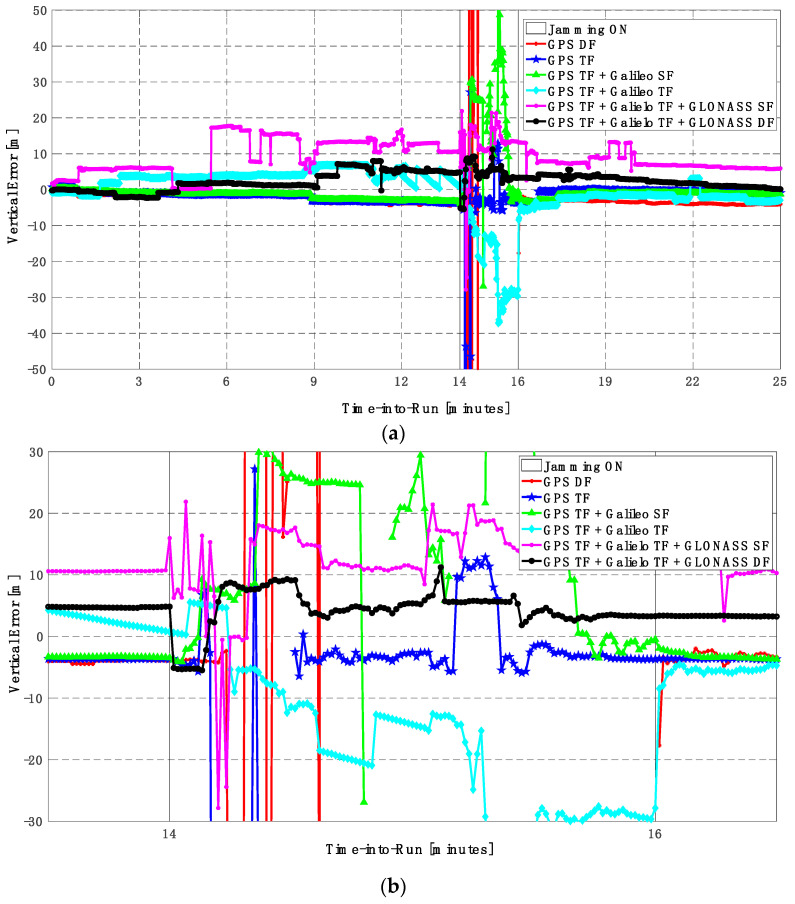
(**a**) Vertical positioning error for different GNSS signal combinations [first trajectory]. (**b**) Zoomed view of vertical positioning error for different GNSS signal combinations [first trajectory].

**Figure 22:**
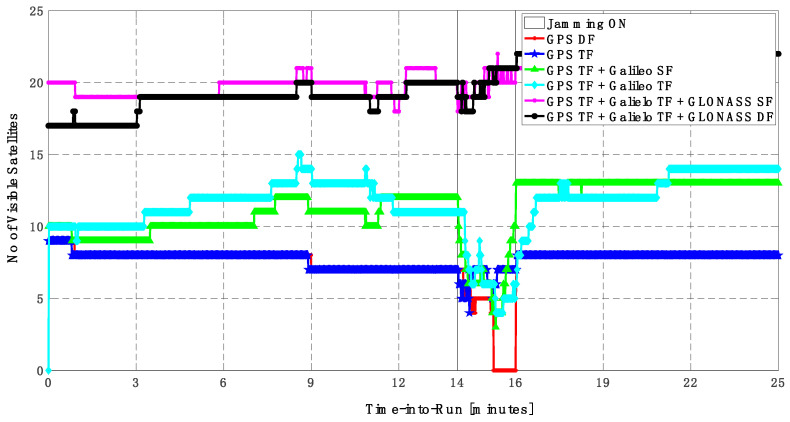
Number of visible satellites for different GNSS signal combinations [first trajectory].

It can be seen from Figure 20a,b and Figure 21a,b that using only the GPS DF signals fails to provide a continuous positioning solution during jamming. By using the GPS TF signals or the GPS TF + Galileo SF signals, the positioning solution is still suffering from discontinuity at some epochs where the number of satellites is less than five, as shown in Figure 22. By adding the Galileo TF signals to the GPS TF signals, the performance of the positioning solution is significantly improved. Additionally, adding the GLONASS SF and DF signals to the GPS TF and the Galileo TF signals improves the performance of the positioning solution. This leads to a smoothed positioning solution, especially during jamming intervals, for the GPS TF + Galileo TF + GLONASS DF-based solution with maximum errors of less than 10 m for both horizontal and vertical positioning components, respectively.

From Table 6, adding the Galileo SF signals to the GPS TF signals or adding the GLONASS SF signals to the GPS TF + Galileo TF signals does not improve the positioning solutions for all cases, as the jamming signal is already attacking L1 frequency and degrades the contribution of the included SF signals. Additionally, the root mean square error (RMSE) is improved by about 40% for the horizontal positioning error by using the GPS TF + Galileo TF signals, compared to using only the GPS TF signals. The maximum vertical positioning error decreased dramatically from 163.453 m using the GPS TF signals to 37.169 m using the GPS TF + Galileo TF signals. Moreover, using the GPS TF + Galileo TF + GLONASS DF signals is the only way to have a reliable, accurate, and continuous positioning solution. The RMSE is improved by about 4% and 50% in the horizontal and vertical directions, respectively, by using the GPS TF + Galileo TF + GLONASS DF signals, compared to using the GPS TF + Galileo TF signals. The maximum horizontal and vertical positioning errors decreased from 9.600 m and 37.169 m in the case of using the GPS TF + Galileo TF signals to 5.847 m and 11.220 m in the case of using the GPS TF + Galileo TF + GLONASS DF signals.

#### 6.1.2. Second Trajectory

**Figure 23:**
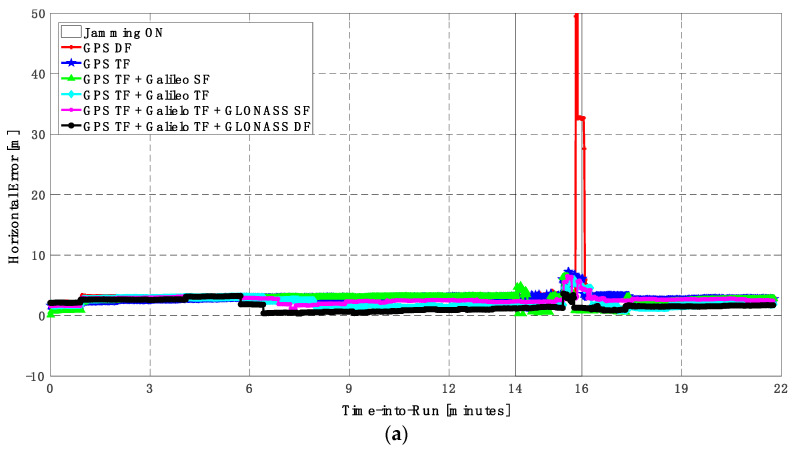

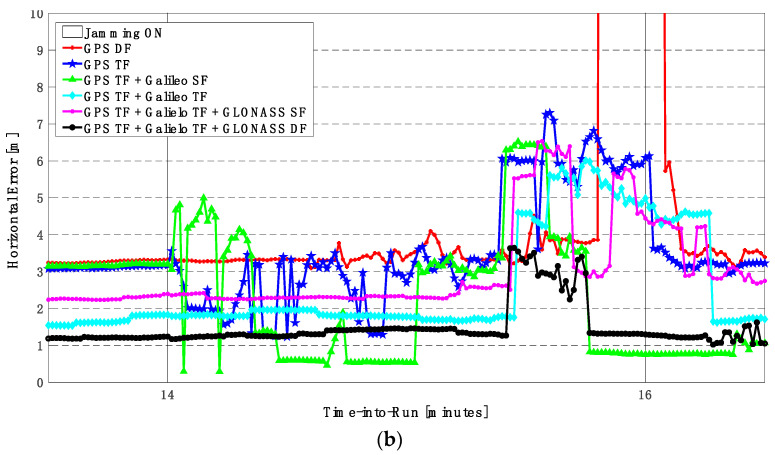
(**a**) Horizontal positioning error for different GNSS signal combinations [second trajectory]. (**b**) Zoomed view of horizontal positioning error for different GNSS signal combinations [second trajectory].

**Figure 24:**
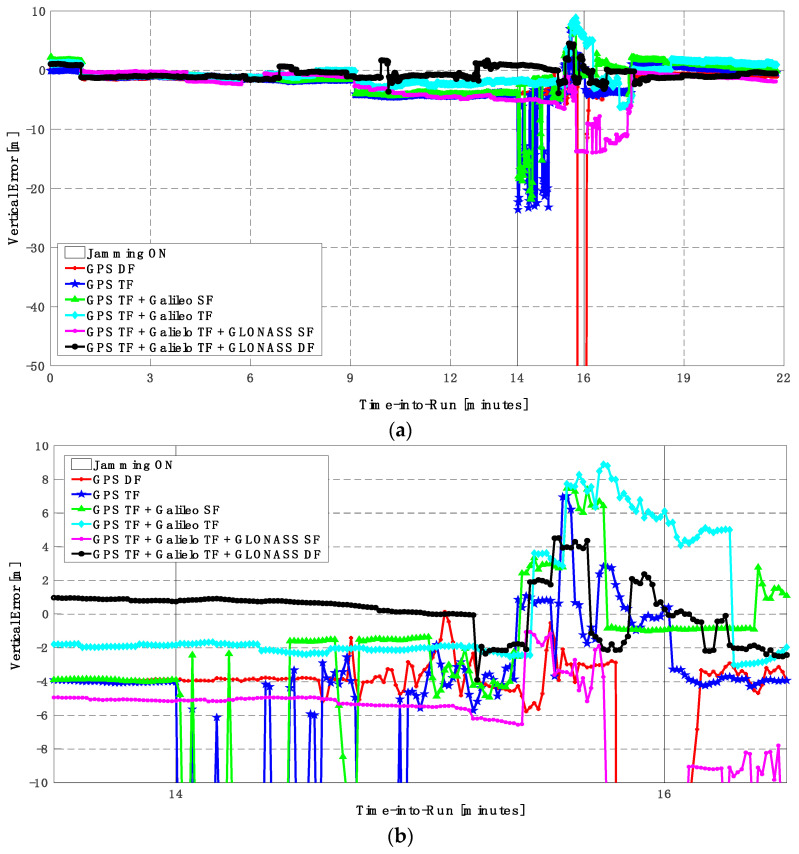
(**a**) Vertical positioning error for different GNSS signal combinations [second trajectory]. (**b**) Zoomed view of vertical positioning error for different GNSS signal combinations [second trajectory].

For this trajectory, the GPS DF-based solution suffers from a partial outage, as shown in Figure 23 and Figure 24. By using the GPS TF signals, the positioning solution performance is slightly improved. Additionally, by using the GPS TF + Galileo SF signals, the positioning solution improves compared to the GPS TF-based solution. By using the Galileo TF signals along with the GPS TF signals, the positioning solution performance is significantly improved compared to the GPS TF counterpart.

**Figure 25:**
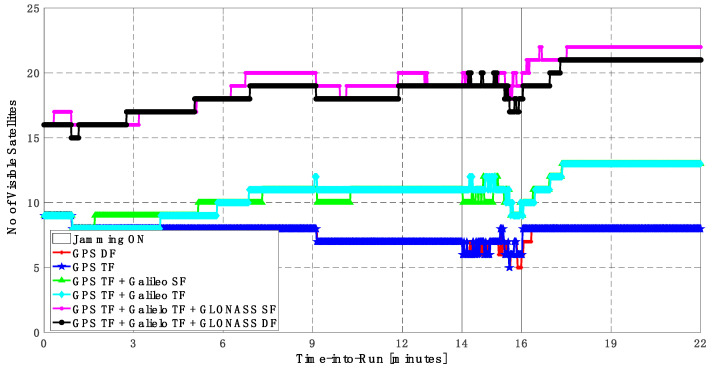
Number of visible satellites for different GNSS signal combinations [second trajectory].

Like the first trajectory, the inclusion of just the GLONASS SF signals does not improve the positioning solution performance. However, adding the GLONASS DF signals to the GPS TF and the Galileo TF signals improves the performance of the positioning solution. This leads to a better positioning solution, especially during jamming intervals, for the GPS TF + Galileo TF + GLONASS DF solution. The main reason is that the number of visible satellites is more than 15 when using GPS, Galileo, and GLONASS signals, compared to 10 when using GPS and Galileo signals, as shown in Figure 25.

The statistical analysis presented in Table 7 indicates that the GPS DF-based positioning solution offers the worst performance. Additionally, the GPS TF-based solution improves positioning performance, compared to its GPS DF counterpart. The GPS TF + Galileo SF-based solution shows a slight improvement compared to the GPS TF counterpart. Moreover, using the GPS TF + Galileo TF signals improves the RMSE of the positioning solution by about 22% and 50% for the horizontal and vertical positioning components, respectively, compared to the GPS TF counterpart. Adding the GLONASS SF signals to the GPS TF signals and Galileo TF signals does not improve the positioning performance compared to the GPS TF + Galileo TF counterpart. This is because of the effect of jamming insertion on L1 frequency. However, using the GLONASS DF signals with both the GPS TF signals and the Galileo TF signals provides a reliable and accurate positioning solution compared to a GPS TF + Galileo TF-based positioning solution. The RMSE improves by about 22% and 44% for the horizontal and vertical positioning components, compared to the GPS TF + Galileo TF-based solution.

### 6.2. High Jamming Scenario

Finally, the performance of the GNSS receiver was assessed under high actual jamming conditions for both trajectories.

#### 6.2.1. First Trajectory

**Figure 26:**
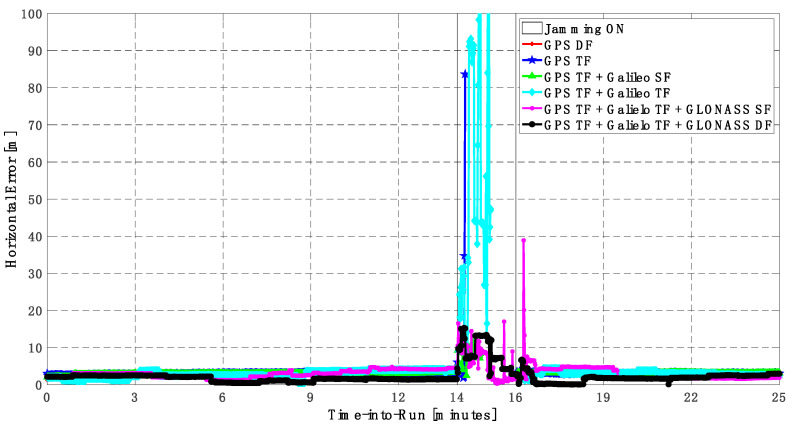
Horizontal positioning error for different GNSS signal combinations [first trajectory].

**Figure 27:**
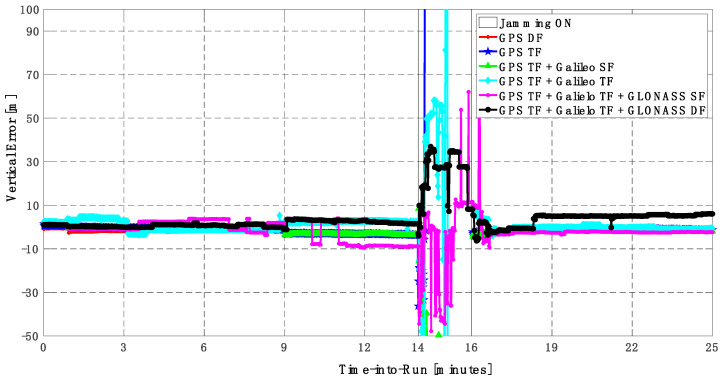
Vertical positioning error for different GNSS signal combinations [first trajectory].

As shown in Figure 26 and Figure 27, the GPS DF, GPS TF, GPS TF + Galileo SF, and GPS TF + Galileo TF-based solutions fail to provide the anticipated continuous positioning solution during jamming intervals. The inclusion of either the GLONASS SF or the GLONASS DF signals leads to a continuous positioning solution during jamming intervals. However, the GPS TF + Galileo TF + GLONASS DF-based solution has superior positioning performance compared to the GPS TF + Galileo TF + GLONASS SF counterpart.

**Figure 28:**
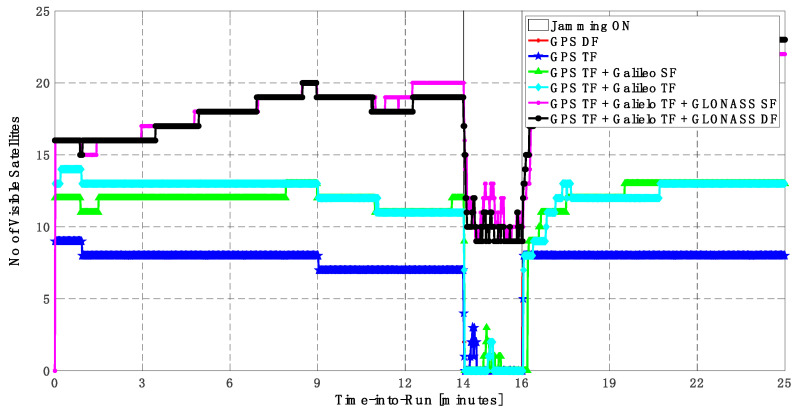
Number of visible satellites for different GNSS signal combinations [first trajectory].

The minimum number of visible satellites using the GPS, Galileo, and GLONASS signals is 9, compared to less than one by using either GPS or GPS + Galileo signals, as shown in Figure 28.

It is clear from Table 8 that the GPS TF-based solution is worse than the GPS DF solution due to the lower availability of the GPS DF compared to the GPS TF counterpart. Similarly, the GPS TF + Galileo TF solution shows worse performance than the GPS TF + Galileo SF counterpart, as the availability of the GPS TF + Galileo SF is less than the GPS TF + Galileo TF counterpart. The most accurate positioning solution is achieved through the GPS TF + Galileo TF + GLONASS DF combination. The RMSE of the GPS TF + Galileo TF + GLONASS DF positioning solution is improved by about 79% and 52%, respectively, compared to the GPS TF + Galileo TF counterpart.

#### 6.2.2. Second Trajectory

**Figure 29:**
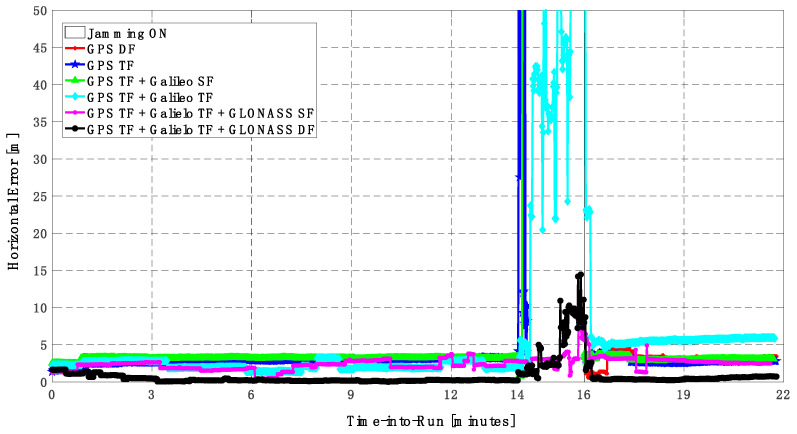
Horizontal positioning error for different GNSS signal combinations [second trajectory].

**Figure 30:**
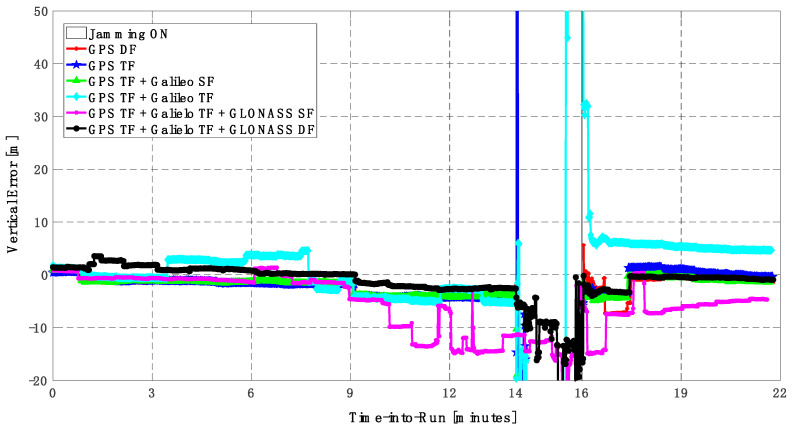
Vertical positioning error for different GNSS signal combinations [second trajectory].

**Figure 31:**
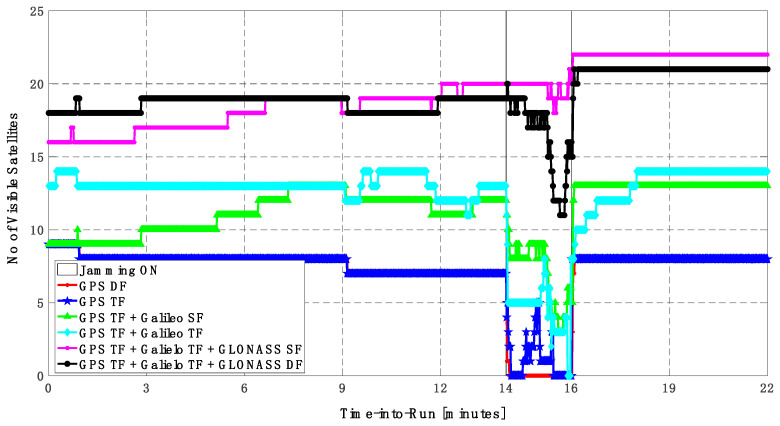
Number of visible satellites for different GNSS signal combinations [second trajectory].

Similar to the first trajectory, the GPS DF, GPS TF, GPS TF + Galileo SF, and GPS TF + Galileo TF-based solutions fail to provide continuous positioning solutions during jamming intervals, as shown in Figure 29 and Figure 30. The solutions that provide a continuous positioning solution during jamming intervals are both the GPS TF + Galileo TF + GLONASS SF and the GPS TF + Galileo TF + GLONASS DF-based solutions, due to the availability of more than 10 satellites during jamming, as shown in Figure 31. Additionally, the GPS TF + Galileo TF + GLONASS DF-based solution provides the best overall positioning performance during the jamming interval. It is clear from Table 9 that the GPS TF-based solution is worse than the GPS DF solution due to the lower availability of the GPS DF compared to the GPS TF counterpart. Likewise, the RMSE of the GPS TF + Galileo TF-based solution is worse than the GPS TF + Galileo SF counterpart, as the availability of the GPS TF + Galileo SF is less than the GPS TF + Galileo TF counterpart.

The most accurate positioning solution is achieved through the GPS TF + Galileo TF + GLONASS DF combination. As shown in Table 9, the RMSE of the GPS TF + Galileo TF + GLONASS DF solution is improved by about 86% and 77%, compared to the GPS TF + Galileo TF counterpart.

## 7. Discussion

**Figure 32:**
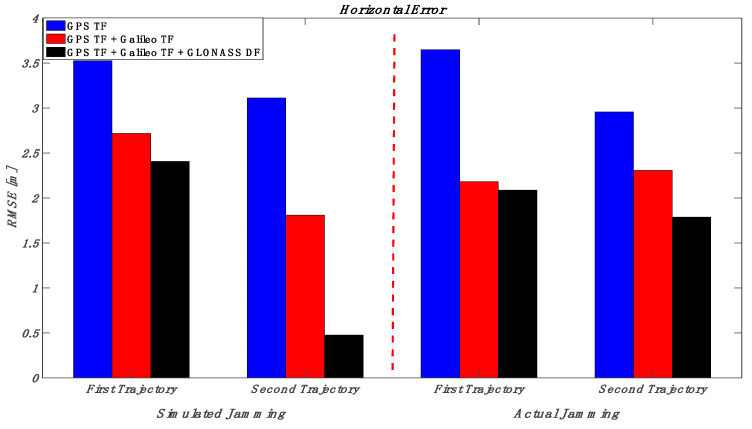
RMS of horizontal errors for the simulated and actual jamming scenarios (low jamming).

**Figure 33:**
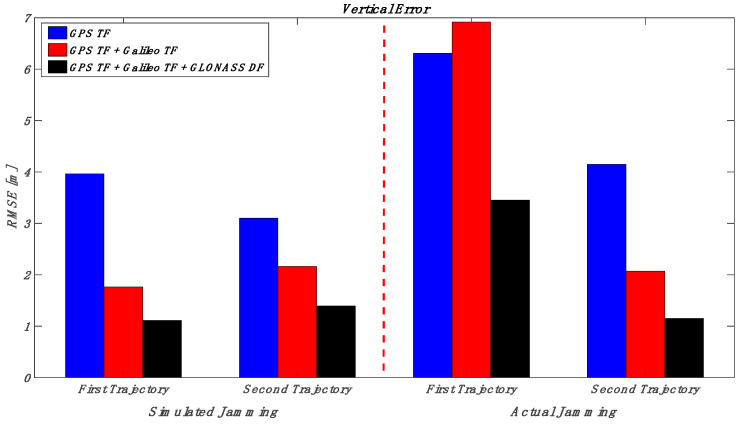
RMS of vertical errors for the simulated and actual jamming scenarios (low jamming).

**Figure 34:**
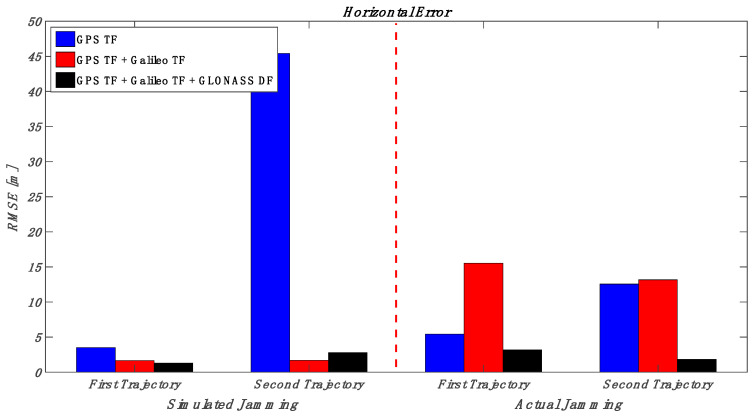
RMS of horizontal errors for the simulated and actual jamming scenarios (high jamming).

**Figure 35:**
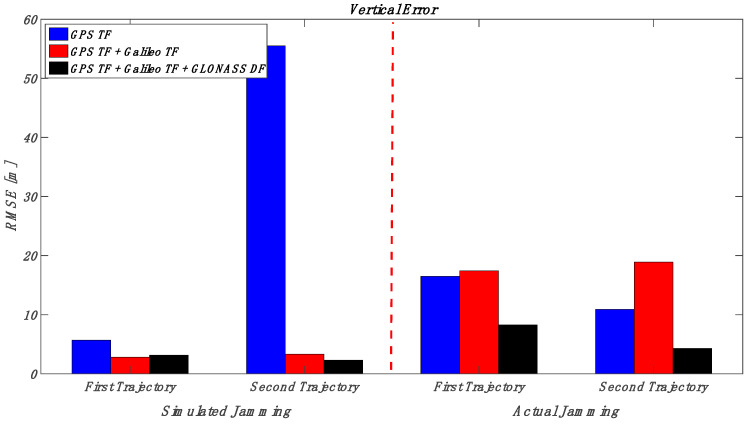
RMS of vertical errors for the simulated and actual jamming scenarios (high jamming).

This section puts together the obtained results for those various multi-frequency, multi-constellation GNSS signal groupings; jamming signal type and jamming power level; and trajectory dynamics. Looking at Figure 32 and Figure 33 (the low power case for both simulated and actual jamming), it is obvious that the performance of the GNSS receiver in the second trajectory is much better than the first trajectory, especially for the actual jamming, due to the lower dynamics compared to the first trajectory. Additionally, the multi-frequency GPS signal-based positioning solution is barely affected by the applied low-power jamming signal. As an example, the maximum RMSE is about 6 m in the vertical positioning component for the first trajectory under an actual jamming attack. By combining multi-frequency signals from both GPS and Galileo, the positioning shows better performance in both horizontal and vertical positioning errors. However, the RMSE of the GPS TF + Galileo TF-based solution in the vertical direction is slightly worse than the GPS TF counterpart in the first trajectory. This is because the number of Galileo satellites decreased suddenly, as shown in Figure 22, especially during jamming, affecting the resultant solution performance in the vertical direction, as presented before in Figure 21. Moreover, the more constellations incorporated in the positioning solution, the less the RMSE is, leading to the best solution derived from the GPS, Galileo, and GLONASS constellations combined. In the high jamming case, as shown in Figure 34 and Figure 35, the GPS-only solution was the worst case, which accumulated position errors during the jamming period that grew up to about 45 m in the horizontal component and about 50 m in the vertical component in the simulated jamming case. The RMSE of the same signal combination was better with the real jamming case because the GPS TF-based solution suffered from discontinuities during the jamming interval compared to the full availability of both the GPS + Galileo and GPS + Galileo + GLONASS counterparts during the jamming interval. As a result, the GPS + Galileo and the GPS + Galileo + GLONASS-based solutions have some spikes under high jamming, leading to higher RMS error values compared to the GPS-only solution. The multi-constellation-based positioning solutions maintained better positioning performance even under the high-power jamming signal, which reflects the importance of using a multi-frequency multi-constellation GNSS receiver, especially with high jamming attacks.

## 8. Conclusions

Performance verification of GNSS receivers under signal jamming attacks is never up to researchers’ or users’ satisfaction. The more experiments are conducted, the more knowledge is gained about the issue. This paper presented fresh experiments performed with state-of-the-art GNSS equipment and simulation tools to assess the effect of multi-frequency, multi-constellation GNSS signals on improving the GNSS receiver performance under different signal jamming conditions. First, the contribution of using GPS multi-frequency measurements on the receiver performance was assessed by enabling a dual-frequency (DF) signal at a time, then enabling GPS triple-frequency (TF) signals. The benefit of multi-constellation GNSS signal inclusion to the GNSS receiver performance was evaluated by enabling GPS TF signals and Galileo single-frequency (SF) signals at a time, then enabling GPS TF signals and Galileo TF signals all at once. After that, the GNSS receiver was assessed by enabling GPS TF, Galileo TF, and GLONASS SF signals at a time and then enabling GPS TF signals, Galileo TF signals, and GLONASS DF signals all at once. Additionally, the effect of both simulated and real jamming signals on GNSS signals was examined at different jamming power levels. To further assure the accuracy of the experiments, two airborne trajectories were created with different dynamics. The simulation was completed using the powerful Orolia GSG-8 GNSS signal simulator. The simulated jamming signals were also inserted using the same tool. On the other hand, the real jamming signals were generated using the NavWar Electronic Attack Trainer (NEAT) jammer by NovAtel. The research concluded that using a multi-frequency GPS-only receiver can maintain, to a good extent, a reliable positioning solution. Moreover, a multi-constellation GNSS receiver can maintain a continuous, robust positioning solution even under high power jamming conditions. There were always more than a dozen visible satellites, regardless of the type/level of applied jamming signal, when enabling the latter receiver feature. This allowed for open-sky-like positioning accuracy.

## Figures and Tables

**Table 1:** GNSS combinations.

Combination	GNSS Constellation	Signals Acquired
1	GPS DF	L1 C/A, L1P, L2C, L2P
2	GPS TF	L1 C/A, L1P, L2C, L2P, L5
3	GPS TF + Galileo SF	L1 C/A, L1P, L2C, L2P, L5, E1
4	GPS TF + Galileo TF	L1 C/A, L1P, L2C, L2P, L5, E1, E5a, E5b
5	GPS TF + Galileo TF + GLONASS SF	L1 C/A, L1P, L2C, L2P, L5, E1, E5a, E5b, G1
6	GPS TF+ Galileo TF+ GLONASS DF	L1 C/A, L1P, L2C, L2P, L5, E1, E5a, E5b, G1, G2

**Table 2:** RMS error, mean, and maximum (max) positioning errors with low jamming [first trajectory].

Combination	Horizontal			Vertical		
	RMSE	Mean	Max	RMSE	Mean	Max
GPS DF	48.434	6.560	1141.527	147.512	13.840	3283.053
GPS TF	3.528	3.482	8.156	3.964	2.777	31.026
GPS TF + Galileo SF	3.294	3.272	8.187	2.746	1.980	23.401
GPS TF + Galileo TF	2.720	2.678	4.624	1.766	1.578	4.624
GPS TF+ Galileo TF + GLONASS SF	2.853	2.825	4.851	1.845	1.687	4.851
GPS TF + Galileo TF + GLONASS DF	2.409	2.384	3.074	1.115	0.980	4.024

**Table 3:** RMS error, mean, and Max positioning errors with low jamming [second trajectory].

Combination	Horizontal Error			Vertical		
	RMSE	Mean	Max	RMSE	Mean	Max
GPS DF	2.994	2.949	9.882	3.264	2.734	17.911
GPS TF	3.112	3.084	6.139	3.103	2.173	16.388
GPS TF + Galileo SF	3.154	3.138	4.914	2.545	2.128	22.633
GPS TF + Galileo TF	1.810	1.544	4.517	2.163	1.647	10.969
GPS TF+ Galileo TF + GLONASS SF	1.243	1.024	4.390	3.163	2.280	13.842
GPS TF + Galileo TF + GLONASS DF	0.477	0.391	2.596	1.394	1.067	6.393

**Table 4:** RMS error, mean, and Max positioning errors with high jamming [first trajectory].

Combination	Horizontal			Vertical		
	RMSE	Mean	Max	RMSE	Mean	Max
GPS DF	45.243	10.621	344.106	132.595	25.015	1026.897
GPS TF	3.492	3.261	28.611	5.701	2.732	94.982
GPS TF + Galileo SF	4.045	3.377	48.045	10.212	3.474	158.456
GPS TF + Galileo TF	1.670	1.333	8.904	2.809	1.799	13.211
GPS + Galileo TF + GLONASS SF	2.179	1.649	18.975	4.324	2.271	47.251
GPS TF + Galileo TF + GLONASS DF	1.300	1.110	3.347	3.166	2.274	11.363

**Table 5:** RMS error, mean, and Max positioning errors with high jamming [second trajectory].

Combination	Horizontal			Vertical		
	RMSE	Mean	Max	RMSE	Mean	Max
GPS DF	39.087	6.750	841.198	87.420	12.630	1757.638
GPS TF	45.413	6.217	1001.927	55.505	5.340	1272.265
GPS TF + Galileo SF	3.225	3.212	4.923	2.762	2.104	15.265
GPS TF + Galileo TF	1.707	0.975	7.245	3.332	2.285	13.756
GPS TF+ Galileo TF + GLONASS SF	2.015	1.781	11.262	2.314	1.580	18.614
GPS TF + Galileo TF + GLONASS DF	2.800	2.754	5.137	2.330	1.507	11.658

**Table 6:** RMS error, mean, and Max positioning errors with low actual jamming [first trajectory].

Combination	Horizontal			Vertical		
	RMSE	Mean	Max	RMSE	Mean	Max
GPS DF	177.205	24.096	3908.975	328.590	51.193	4711.388
GPS TF	3.649	3.166	56.892	6.308	2.022	163.453
GPS TF + Galileo SF	4.123	3.586	15.631	6.482	2.747	98.680
GPS TF + Galileo TF	2.182	1.996	9.600	6.917	4.481	37.169
GPS TF+ Galileo TF + GLONASS SF	4.204	2.865	33.975	10.308	9.223	27.846
GPS TF + Galileo TF + GLONASS DF	2.089	1.937	5.847	3.454	2.810	11.220

**Table 7:** RMS error, mean, and Max positioning errors with low actual jamming [second trajectory].

Combination	Horizontal			Vertical		
	RMSE	Mean	Max	RMSE	Mean	Max
GPS DF	6.079	3.587	114.251	15.068	3.660	330.936
GPS TF	2.958	2.881	7.301	4.152	2.517	23.592
GPS TF + Galileo SF	2.721	2.573	6.517	3.425	2.265	21.818
GPS TF + Galileo TF	2.307	2.155	6.009	2.070	1.625	8.898
GPS TF+ Galileo TF + GLONASS SF	2.693	2.622	6.534	4.143	2.814	13.929
GPS TF + Galileo TF + GLONASS DF	1.787	1.585	3.640	1.151	1.014	4.519

**Table 8:** RMS error, mean, and Max positioning errors with high actual jamming [first trajectory].

Combination	Horizontal			Vertical		
	RMSE	Mean	Max	RMSE	Mean	Max
GPS DF	2.845	2.825	3.569	2.278	1.891	4.613
GPS TF	5.419	3.202	134.165	16.495	2.435	407.822
GPS TF + Galileo SF	3.280	3.137	11.383	9.548	3.561	61.381
GPS TF + Galileo TF	15.542	5.289	251.315	17.454	4.531	273.599
GPS TF+ Galileo TF + GLONASS SF	3.749	3.102	38.859	7.933	4.471	68.720
GPS TF + Galileo TF + GLONASS DF	3.200	2.254	15.289	8.299	4.327	36.932

**Table 9:** RMS error, mean, and maximum (Max) positioning errors with high actual jamming [second trajectory].

Combination	Horizontal			Vertical		
	RMSE	Mean	Max	RMSE	Mean	Max
GPS DF	3.211	3.173	4.541	2.673	2.196	7.453
GPS TF	12.559	3.638	356.038	10.902	2.940	187.952
GPS TF + Galileo SF	12.272	4.201	138.110	11.368	3.195	124.674
GPS TF + Galileo TF	13.161	6.455	58.532	18.938	8.636	122.068
GPS TF+ Galileo TF + GLONASS SF	2.575	2.420	8.316	7.827	5.735	42.224
GPS TF + Galileo TF + GLONASS DF	1.831	0.781	14.4477	4.296	2.343	23.135

## References

[1] Purfürst T. (2022). Evaluation of Static Autonomous GNSS Positioning Accuracy Using Single-, Dual-, and Tri-Frequency Smartphones in Forest Canopy Environments. Sensors.

[2] Li X., Ge M., Dai X., Ren X., Fritsche M., Wickert J., Schuh H. (2015). Accuracy and reliability of multi-GNSS real-time precise positioning: GPS, GLONASS, BeiDou, and Galileo. J. Geod..

[3] Joubert N., Reid T.G.R., Noble F. Developments in Modern GNSS and its Impact on Autonomous Vehicle Architectures. Proceedings of the 2020 IEEE Intelligent Vehicles Symposium (IV).

[4] Dasgupta S., Rahman M., Bandi T.N. AI-based GNSS Spoofing Attack Detection for Autonomous Vehicles using Satellite Characteristics Data. Proceedings of the 2023 International Technical Meeting, ION ITM 2023.

[5] Jing H., Gao Y., Shahbeigi S., Dianati M. (2022). Integrity Monitoring of GNSS/INS Based Positioning Systems for Autonomous Vehicles: State-of-the-Art and Open Challenges. Trans. Intell. Transp. Syst..

[6] Lin X., Wang F., Yang B., Zhang W. (2021). Autonomous Vehicle Localization with Prior Visual Point Cloud Map Constraints in GNSS-Challenged Environments. Remote Sens..

[7] Schütz A., Sánchez-Morales D.E., Pany T. Precise Positioning Through a Loosely-coupled Sensor Fusion of GNSS-RTK, INS and LiDAR for Autonomous Driving. Proceedings of the 2020 IEEE/ION Position, Location and Navigation Symposium (PLANS).

[8] Chen Y., Zhan X. (2021). GNSS vulnerability reliable assessment and its substitution with visual–inertial navigation. Aerosp. Syst..

[9] Lo S., Chen Y.H., Miguel N.S., Walter T. Examining Cross Frequency Interference Effects in Multi-Frequency GNSS Receivers. Proceedings of the 2023 International Technical Meeting, ION ITM 2023.

[10] Kaplan E.D., Hegarty C. (2017). Understanding GPS/GNSS: Principles and Applications.

[11] ElGhamrawy H. (2019). Narrowband Jamming Mitigation in Vector-Based GPS Software Defined Receiver. Ph.D. Thesis.

[12] Elghamrawy H., Karaim M., Korenberg M., Noureldin A. (2022). High-Resolution Spectral Estimation for Continuous Wave Jamming Mitigation of GNSS Signals in Autonomous Vehicles. IEEE Trans. Intell. Transp. Syst..

[13] Reuper B., Becker M., Leinen S. (2018). Benefits of Multi-Constellation/Multi-Frequency GNSS in a Tightly Coupled GNSS/IMU/Odometry Integration Algorithm. Sensors.

[14] Dong Y., Wang D., Zhang L., Li Q., Wu A. (2020). Tightly Coupled GNSS/INS Integration with Robust Sequential Kalman Filter for Accurate Vehicular Navigation. Sensors.

[15] Li X., Shen Z., Li X., Liu G., Zhou Y., Li S. (2023). Continuous Decimeter-Level Positioning in Urban Environments Using Multi-Frequency GPS/BDS/Galileo PPP/INS Tightly Coupled Integration. Remote Sens..

[16] Ollander S. (2020). Accurate Positioning in Urban Canyons with Multi-Frequency Satellite Navigation. Ph.D. Thesis.

[17] Elghamrawy H., Karaim M., Noureldin A., Tamazin M. (2020). Experimental Evaluation of the Impact of Different Types of Jamming Signals on Commercial GNSS Receivers. Appl. Sci..

[18] Reuper B.F. (2020). Multi-Frequency GNSS Sensor Fusion with Quality Assessment for Automotive 583 Applications. Ph.D. Thesis.

[19] Elmasry O., Tamazin M., Elghamrawy H., Karaim M., Noureldin A., Khedr M. Examining the benefits of multi-GNSS constellation for the positioning of high dynamics air platforms under jamming conditions. Proceedings of the 11th International Symposium on Mechatronics and its Applications (ISMA).

[20] Zidan J., Adegoke E.I., Kampert E., Birrell S.A., Ford C.R., Higgins M.D. (2021). GNSS Vulnerabilities and Existing Solutions: A Review of the Literature. IEEE Access.

[21] Glomsvoll O., Bonenberg L.K. (2018). GNSS Jamming Resilience for Close to Shore Navigation in the Northern Sea. J. Navig..

[22] Bhuiyan M.Z.H., Honkala S., Soderhölm S., Kuusniemi H. Performance analysis of a multi-GNSS receiver in the presence of a commercial jammer. Proceedings of the 2015 International Association of Institutes of Navigation World Congress (IAIN).

[23] Safran Safran-Navigation-Timing. https://safran-navigation-timing.com/manuals/skydel/#_launching_skydel.

[24] NovAtel NovAtel-PwrPak7. NovAtel. https://chrome-extension://efaidnbmnnnibpcajpcglclefindmkaj/https://hexagondownloads.blob.core.windows.net/public/Novatel/assets/Documents/Papers/PwrPak7-Product-Sheet/PwrPak7-Product-Sheet.pdf.

